# An analysis of child protection ‘standard operating procedures for research’ in higher education institutions in the United Kingdom

**DOI:** 10.1186/s12910-015-0058-0

**Published:** 2015-09-29

**Authors:** Duncan Randall, Kristin Childers-Buschle, Anna Anderson, Julie Taylor

**Affiliations:** University of Southampton, Southampton, UK; University of Edinburgh/NSPCC Child Protection Research Centre, Edinburgh, UK; School of Health and Population Science, University of Birmingham, Edgbaston, Birmingham, B15 2TT UK

**Keywords:** Research ethics, Child maltreatment, Child protection, Standard operating procedures

## Abstract

**Background:**

Interest in children’s agency within the research process has led to a renewed consideration of the relationships between researchers and children. Child protection concerns are sometimes not recognised by researchers, and sometimes ignored. Yet much research on children’s lives, especially in health, has the potential to uncover child abuse. University research guidance should be in place to safeguard both researchers and the populations under scrutiny. The aim of this study was to examine university guidance on protecting children in research contexts.

**Methods:**

Child protection Standard Operating Procedures (SOPs) were requested from institutions with Research Assessment Exercise (2008) profiles in the top two quartiles according to published league tables. Procedures were included if they applied across the institution and if they were more extensive than stating the university’s general application of the UK Disclosure and Barring Service process. A typology for scoring the SOPs was designed for this study based on the authors’ previous work. The typology and the raw data scoring were reviewed independently by each of the team members and collectively agreed. The raw scores were charted and analysed using descriptive statistics.

**Results:**

SOPs for research conduct amongst vulnerable groups were sought from 83 institutions. Forty HEIs provided policies which met the inclusion criteria. The majority did not mention children, young people or vulnerable adults as a whole, although children in nurseries and young people in universities were addressed. Only three institutions scored over 50 out of a possible 100. The mean score was 17.4. More than half the HEIs made no reference to vetting/barring schemes in research, only eight universities set out a training programme on child protection. Research was often not mentioned in the SOPs and only six mention children in research, with only two fully recognising the extent of child protection in research.

**Discussion:**

There is potential for researchers to recognise and respond to maltreatment of children who participate in research. However, the majority of HEIs do not have an overt culture of safeguarding. There is confusion over what are the roles and responsibilities of HEIs in relation to research that involves children.

**Conclusions:**

The policies that are meant to support and guide research practice, so that children are protected, are in the most part non-existent or poorly developed.

## Background

Maltreatment (abuse and neglect) of children is a substantial and serious concern. Although definitive figures for the prevalence of child abuse are unknown, current research suggests that for every one child that is known to child protection agencies another eight children are potentially experiencing maltreatment, but are unknown to these agencies in the United Kingdom (UK) [1]. Reporting maltreatment is often vague in law although everyone has a responsibility in this regard. Worldwide we know that violence against children is at epidemic levels [2]. In the past few years there has been an increasing willingness to listen to children as abuse victims and to prosecute perpetuators of historical child abuse [3]. Public concern has been heightened by high profile cases such as the Savile case [4], those concerning Roman Catholic priests [5] and the inquiry into the Salvation Army children’s homes in Australia [6]. Alongside this increasing public awareness there has been a shift in how researchers address children’s needs within a research context, whereby the focus has moved from research *on* children to research *with* children [7]. This distinction represents a change from primarily collecting data about children from adults, to now often involving children in participatory research. As research *with* children has increased over the last decade significant advancements in the social and political lives of children have developed simultaneously, allowing an improved understanding of the role children can play within all forms of societal structures. Cater and Overlein [8] argue that to develop practice researchers need to involve children in their research and to focus on children’s collectively shared experiences to improve children’s services and lives. This move to value children’s social experience and to participatory research allows for not only an increased multidimensional understanding of the lives of children, but also a shift in the relationship between researchers and children [9]. The trust created between researcher and child can be extremely positive for both parties, it can also open doors for the facilitation of disclosure, as well as recognition, of abuse. The increased use of participatory methods as well as the increased focus on aspects of children’s lives previously overlooked (self-harm, for example), together with the development of large cohort studies collecting data sets, stored and analysed using computers, has all increased the opportunities for researchers working with children and their adult carers to uncover abusive behaviours.

Some children are particularly vulnerable to abuse, for example, disabled children, those with chronic or long-term conditions, or those living with parents under the influence of substances or who have severe mental illness [10, 11]. These children are often the very focus of health and social care research. Professionals involved in all forms of research, whether directly related to children or not, have the potential of becoming enmeshed within child protection issues. For example, a research project on mental health issues or substance abuse may focus on adults who are responsible for the care of children. It therefore becomes imperative for researchers to be knowledgeable in how to recognise and report suspected cases of abuses, as well as what to do if a participant discloses that a child could be in danger regardless of whether the children are the original subject of the research.

Randall et al.’s 2013 [12] review of current literature on the child protection in research contexts demonstrated that the approach taken by research teams regarding protecting children in research contexts was highly variable. Within the literature there were suggestions and discussions of how to improve the safety of children in research by: accessing training for researchers in child protection (or employing researchers with a professional background that included child protection training); making protection processes transparent; consulting children on how to deal with abuse issues within the research encounter; and finally establishing links with existing child protection services [12].

Randall et al. [12] noted these measures would be in addition to ethical review, but that the complexities of dealing with child protection could not be addressed by a single point of ethical opinion; rather it was suggested an ethic of care is required in which researchers can access continual supervision and advice from experienced child protection workers during projects. Previous research on sensitive topics suggests that while laws and ethical codes may guide how researchers behave in research contexts “they may be insufficient to cover complex situations and may conflict or be hard to interpret” [13]. Single point ethical guidance may therefore be inadequate, such as with child abuse, where there is a blurring of lines between protection and confidentiality. Frameworks focusing on maintaining ethical research with children have constantly targeted the balance between researcher’s aims while simultaneously protecting participants [14]. Such frameworks have their roots in the United Nations Convention on the Rights of a Child which sets out child-specific needs and rights [15]. Although no specific article references the rights of children in research, there is much that can be inferred from article 3 paragraph 1: “in all actions concerning children, whether undertaken by public or private social welfare institutions, courts of law, administrative authorities, or legislative bodies, the best interests of the child should be a primary consideration” (Article 3, paragraph 1 [see also Articles 12.1 and 13.1 [15]]). Although policies and procedures often suggest that researchers report cases of suspected or disclosed abuse, such individuals may be unsure to whom exactly they should report. More crucially in situations where a child could, or even does, disclose possible abuse, researchers are not mandated in all countries by formal law to report suspected abuse. Although there is some pressure in England to introduce a mandatory reporting law, studies from other countries have not shown this provides better for protection for children [12]. More disquieting was the evidence uncovered by Randall et al. [12] of researchers not only avoiding reporting the abuse of children, but also deliberately constructing research protocols to obscure abuse. Such shielding of abuse could potentially lead to an escalation of abuse and a lifetime of abuse against a child. Whilst mandatory reporting is not regulatory in the UK, suspicions of child abuse need to be addressed for the safety and protection of children, as well as researchers.

Voluntary organisations offer guidelines for professionals regarding what to do if a participant discloses abuse and how to shape research agendas around protecting children [16]. Although several pieces of legislation over the years in the UK have attempted to address the relationship between various professional groups and child protection, the majority have fallen short of defining the specific role of researchers and Higher Education Institutions (HEIs) in child protection. The national English guidance ‘Working Together to Safeguard Children’, for example, outlines the roles and responsibilities of a wide range of organisations that work with children, including governmental and educational organisations; however, the guidance makes no mention of research professionals [17, 18]. Additional guidance for England in 2007 for HEIs recommended the writing of policies and procedures and additional education for staff, but in relation to education provision not research activities [19]. This guidance also does not suggest what the policy should include or how it should be structured, implemented, supported, resourced and monitored. Randall et al. [12] review of the literature on protecting children in research did not identify any studies that evaluate policy in higher education. The lack of national standard guidance on addressing disclosure of child abuse in research settings, and the variability of child protection practice and procedure on a national level (i.e. definitions, thresholds and operations) makes research institutions critical establishments for clarifying proper procedures for researchers. The study reported here builds on Randall et al. [12] work to attempt to understand if the practices reported in the literature are reflected in the Standard Operating Procedures (SOPs) of research focused universities in the UK.

## Methods

This study was an inductive review of Higher Education Institutes (HEI) Standard Operating Procedures (SOPs) relating to safeguarding children, young people and vulnerable adults. The relevant university ethics procedures were followed. The research was desk-based without participants and under the four-tier system applied at the University of Edinburgh fell under level 0 – no obligation for ethics review. However, we have anonymised data so as not to identify any particular institution.

HEIs were selected from the top 200 UK universities in the Complete University Guide [20] and the Times Higher Education college rankings [21]. The Complete University Guide [20] is compiled by Mayfield University Consultants and uses nine criteria, including research assessment/quality which measures average quality of research at a university, to rank UK universities. The Times Higher Education University Rankings [21] is powered by Thomson Reuters and is the only global university performance table to judge world-class HEIs across core missions (teaching, research, knowledge, international outlook). Rankings were examined for the top 200 HEIs in the UK for the year 2013. From these 200 HEIs the Research Assessment Exercise (RAE) score for each university was examined and collected for inclusion. The RAE score is a measure of average quality of research undertaken at the university level and is undertaken every five years in the UK. The maximum score for RAE (2008) was 4.00. For this study, HEIs with an RAE score of 2.00 or higher were included (i.e. the upper two quartile scores). Although some research-orientated universities opt out of the assessment procedures these HEIs were independently scored with a ‘statistical transformation of (HESA) staff data’ by the Higher Educational Statistical Agency (HESA), thus enabling them to be scored by the Times Higher Education ranking and included in this study. Using these criteria 83 of the top performing research active HEIs were included. This method is of course biased towards the best research led universities.

SOPs for research were obtained through contacting the human resources departments of the HEIs included. Individual HEIs have been anonymised in this study and allocated a research number.

**Fig. 1:**
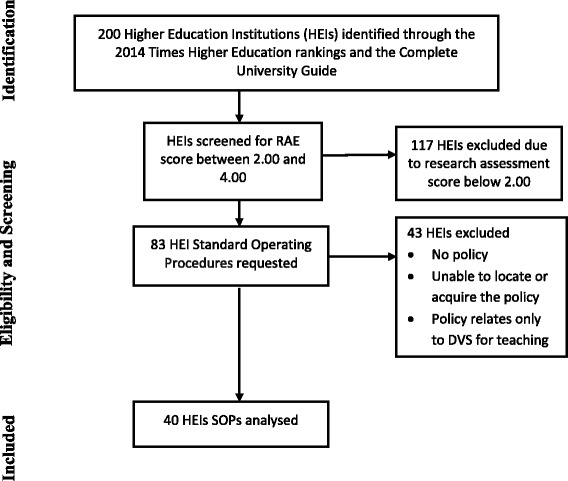
Inclusion/exclusion criteria

HEIs were excluded from analysis if their SOPs only described a barring and vetting scheme, or if the SOP was not applicable university-wide; this criterion affected a total of 28 HEIs. SOPs were examined for mention of children, young people or vulnerable adults. The majority did not mention these as a whole, referring to children in nurseries or young adults at universities. Three HEIs declared that they did not have a child protection policy in place at all. Two HEIs were in the process of updating their procedures at the time of analysis and were therefore not available; these were subsequently excluded. Ten policies could not be located by the relevant institutions. Overall of the 83 HEIs eligible on RAE and ranking scores 40 HEIs had policies that were included in the analysis. Figure 1 show the inclusion and exclusion criteria for HEIs in the study.

The scoring typology used in this study was developed for specific use in this study based on previous work undertaken by the authors (DR and JT [12]). A new scoring criterion was deemed necessary due to a lack of relevant scoring schemes available for assessing child protection in SOPs based on previous research and recommendations [12]. The tool was reviewed, focusing on content validity by all members of the team both independently and collectively. All members of the research team independently checked the scoring of a sample of the primary data. The independent scoring was then compared and agreed as a team and interpretations explored. No significant divergent interpretations of the scoring were uncovered.

The scoring for this study was analysed in two sections. First, child protection culture was assessed as set out in Table 1 (C1-C6), where arguments for the policy, background on legislation, safeguarding issues, and roles and responsibilities of staff were examined. Second the research culture was analysed, in which research-orientated policies were examined for specific child protection guidance, including training for researchers, what to do in case of disclosure, and minimising risk to both children andresearchers (see Table 2, R1-R9). Each criterion was weighted with a score based on the importance of the factor in addressed safeguarding issues. Criteria were given a maximum mark score of 6, 12, or 24 depending on their overall significance (for example detailing the action to be taken when reporting abuse was given the highest priority maximum score of 24 [criteria R5, Table 2]). Each criterion was scored according to its weighting either: *full*, *moderate*, *limited*, or *not met*.

**Table 1:** Findings- culture of safeguarding SOP

Criteria	Weighting ( out of )	Full marks	Moderate	Limited	Not met
		n	n	n	n
*C1*: Is an argument set out in the policy for why a safeguarding policy is required and/or desirable?	6	33/40	3/40	3/40	1/40
*C2*: Does the policy give a background on legislation relating to safeguarding children? (nation-specific)	12	7/40	3/40	4/40	26/40
*C3*: Does the policy give definitions of safeguarding issues that children, young people and vulnerable adults may face?	6	19/40	6/40	9/40	6/40
*C4*: Does the policy define the roles and responsibility of staff in relation to safeguarding?	12	17/40	5/40	14/40	4/40
*C5*: Is there a vetting and/or barring scheme in place?	12	16/40	0/40	1/40	23/40
*C6*: Does the policy refer to a training and/or education programme on safeguarding?	12	8/40	8/40	12/40	12/40

Total maximum score of 60 for Culture criteria (C1-6)

**Table 2:** Findings- culture of research SOP

Criteria	Weighting (out of )	Full marks	Moderate	Limited	Not met
		n	n	n	n
*R1*: Does the research SOP relate to the safeguarding culture SOP in the HEI	12	6/40	1/40	5/40	28/40
*R2*: Does the policy identify safeguarding issues in research contexts in which there is an increased risk of identifying safeguarding issues?	6	2/40	1/40	3/40	34/40
*R3*: Does the policy set out moral, ethical, or methodological principles relating to how researchers should address safeguarding issues and/or design research?	6	2/40	1/40	1/40	36/40
*R4*: Does the policy refer to specific training for researchers in safeguarding in research contexts?	24	0/40	2/40	6/40	32/40
*R5*: Does the policy identify action to be taken if abuse is disclosed which may be related to a research participant, or may be concerning people not participating in the research?	24	2/40	2/40	2/40	34/40
*R6*: Does the policy stipulate any safety protocols to be used in research?	12	2/40	1/40	2/40	35/40
*R7*: Is there provision for supervision from senior researchers and/or child protection experts to support researchers in dealing with safeguarding issues?	12	3/40	0/40	1/40	36/40
*R8*: Does the policy require/recommend collaboration with participants and/or child protection services in designing, implementing and/or reviewing research safeguarding protocols and practices?	12	2/40	1/40	1/40	36/40
*R9*: Are the needs of different professional groups who may be involved in research addressed, e.g. professional duty of nurses to report abuse	12	2/40	1/40	0/40	37/40

Total maximum of 120 for Research criteria (R1-9)

No score was given if the assessor felt the criteria was not met (for example in C2, if the policy contained insufficient and/or was missing important legislation). Additionally, SOP’s were scored as limited or moderate if the policy was not clear and concise regarding policies and procedures. A limited score of 2 out of 6, or 4 out of 12, or 6 out of 24 was given depending on the weighting of the criteria. Moderate scores were 4 out of 6, 8 out of 12 and 12 out of 24 respectively. For example in C6 the education factor in culture section might be scored as limited (4 out of possible 12) if the policy mentioned training and/or education programmes for researchers but gave no detail about what the training included or where it could be accessed. The research criteria (R1-9) were evaluated bearing in mind that research might include research with children; research on abuse and/or protection; research with adults who are responsible for the care of children; research on mental health issues, drug and alcohol misuse, and/or domestic abuse; and research with vulnerable adults.

Each institution was given an overall score by combining the culture score and the research score using the following formula (Culture score (60) + (2x research score (120X2 = 240))/3 = 100).

## Results

The results were disaggregated into two categories: scores from the evaluation of the safeguarding culture not specifically related to research contexts; and scores from the analysis of research safeguarding practices sections of each SOP from the included HEIs. Table 2 show a breakdown of the analysis for each section.

**Fig. 2:**
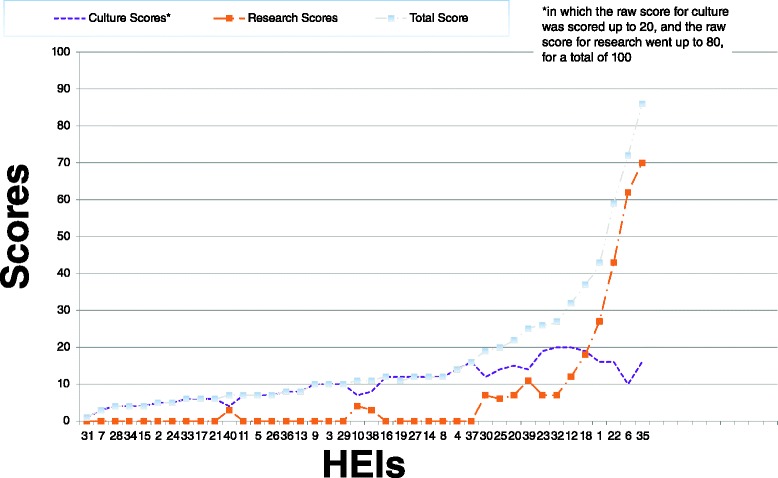
Raw data scores of HEIs

Of the 40 HEIs included in the analysis only three contained policies which addressed the majority of our child protection criteria for research involving children. Scores were weighted out of a possible 100. The highest three scores in the study being 86, followed by 72, and 59. However, the mean score for all HEIs was 17.4. Figure 2 shows a correlation of raw scores for culture and research assessment scores of all HEIs.

While only three HEIs met over half of our criteria, there were several HEIs that had policies in which some issues were addressed more fully than in others.

Only 16 HEIs mentioned a vetting/barring scheme relating to safeguarding children within university environments for staff and students; 23 HEIs made no mention of such schemes. Regarding training and education programmes on safeguarding, only eight HEIs mentioned such programmes in their policies. Twelve HEIs made no reference to training and/or education programmes for staff, eight made moderate reference, and 12 made limited reference. The majority of policies (65 %) lacked sufficient reference to legislative policies relating to the safeguarding of children.

Regarding research specific policies and guidelines the majority of HEI SOPs did not address research contexts at all. Only 12 policies mentioned research and only six made specific mention of children in research. Of the policies that did mention children only two addressed the specific research criteria set out for this study. Only two policies set out moral, ethical, or methodological principles relating to how researchers should address safeguarding issues and/or design research when children are involved (these two policies were the same policies to address specific criteria relating to research with children). There were no policies that received full marks for addressing specific training for researchers in research settings, which included a discussion of safeguarding in relation to research in general. Only two of the 40 policies met, in full, the criteria regarding action to be taken if abuse is disclosed relating to a research participant or concerning people not participating in research. This included adults disclosing information about child abuse regardless if the child and/or adult is a research participant, as well as when the research focus may not be related to children or childhood. Two policies each received moderate and limited marks for this criterion, with 34 policies altogether making no mention of actions to take following disclosure.

## Discussion

### A culture of safeguarding

While the majority of institutions recognise the need for a safeguarding policy few clearly set out the legal position. In England and Wales, for example, if one has reasonable ground to suspect a person is disbarred and undertaking a regulated activity, not referring the person to the Disbarring and Vetting Service is a criminal offence [22] (*Safeguarding Vulnerable Groups Act*, section 18 & 19). While many policies identified the issues in protecting children there seemed to be confusion in identifying who was responsible for taking action. 17 HEIs defined responsibilities while 14 HEIs were vague regarding responsibility for action. This included reporting to the child protection officer with concerns, if the HEI had one in place. The majority of HEIs did not discuss what would constitute a concern or to whom one should report to, thus making determining responsibility incredibly difficult for researchers, potentially placing them in a precarious position. Less than half (16) of the HEIs clearly indicated that they had training programmes on child protection in place for their staff/students. Of these policies, only one provided detail about what that training would incorporate, including information about child protection legislation and policies, how to safeguard children in a variety of university environments- including research- and that the training would be supervised by the local authority child protection team. The remaining 12 policies that did indicate training programmes did not provide detail about what the training would include, only mentioned that the training provision would be internal to the university.

Our analysis of SOPs indicates that there is confusion over what the role and responsibilities of HEIs are in relation to protecting children. Without a strong culture of safeguarding and protecting children it is perhaps not surprising that so few HEIs had a well-developed policy for researching with children. Some institutions appeared only to relate protecting children to their teaching functions indicating that a culture of safeguarding children’s welfare may not always be transferred into research practices.

### Research with children

It is concerning that universities, many of whom are leading the world in children’s research, have for the most part poor or non-existent policies to guide their researchers. It could be argued that such policies are often written only in the wake of a national scandal. The British Broadcasting Corporation and the Catholic Church now have very strong policies [5, 23].The weak policy development indicated in this study perhaps shows that there has been few HEI related scandals to date, which could be interpreted as confirmation of researchers doing a good job of protecting children in research. Alternatively a lack of policies in organisations to protect children could be seen to contribute to cultures which permit and potentially even facilitate abusive behaviours. The lack of policy development and the impression of variable practice uncovered by Randall et al. [12] from their literature review suggest that the research community has neglected to engage in debates on how to protect children in research. This negligence not only raises ethical and moral questions, but also raises methodological issues. The lack of policy on how to conduct research in safer ways enables researchers to design studies which obscure abuse and can be seen as colluding with perpetrators [12]. None of the SOPs we examined for this study set out what action would be taken against researchers who acted in such ways, nor did they set out methodologies and practices recommending how to protect researchers from accusations of abuse.

There is plentiful guidance available on research ethics and working with children [16, 24, 25], but these resources were rarely mentioned in the SOPs. There was no indication in the SOPs of a consensus on how to conduct research into child abuse in ways which might protect children and aid their recovery from abuse.

From this current study we can neither confirm, nor deny that researchers may be appropriately protecting children in the most research intensive universities. All we can say is that the policies which are meant to support and guide research practice, such that children are protected, are in the most part poorly developed and- worryingly- many of these high research performance institutions have no stated policy.

We are in effect relying then on researchers being self-directed. Some researchers who investigate children’s issues are indeed well-qualified and of excellent intention. They may well have accessed training on abuse issues and worked with social care colleagues to gain experience of protecting children. However, it is also possible that they have not had any training and do not uncover abuse against children simply because they do not know where to look or how to recognise signs and cues. Some researchers may not know how to differentiate between varying forms of abuse compared to someone with child protection experience. Certain researchers may have the training and professional background and experience of protecting children in a clinical setting (health visitors for example) but they may not be able to transfer that understanding into a research setting. Researchers may know who to telephone in a clinic if they are concerned, yet few university policies tell them how to report abuse as a researcher. Finally, and most extreme, perpetrators of abuse may use a research role to access children in the absence of effective use of the Disbarring and Vetting Service. In such cases a HEI could be prosecuted for allowing a researcher to undertake a regulated activity such as working with children without having made the necessary checks. Without addressing these potential gaps the safety of children who participate in research is put at risk.

As mentioned in the introduction of this paper, children who are participants in research studies may be at increased risk of potential maltreatment. Often as a society we want to know how to help our most vulnerable citizens and so we undertake research to find ways to improve their lives. These children deserve the highest standards of protection from the research community and no HEIs want to be accused of complacency in such matters. Following the child sexual abuse enquiry of Jerry Sandusky at a major American HEI, Dillinger [26] has demonstrated that 55 of 69 US HEIs had reviewed or revised their policies relating to child abuse prevention and reporting. While reviewed or revised policies to prevent maltreatment are a desired result, such policies should have been already in place.

The lack of a validated tool to measure how Standard Operating Procedures of HEIs address safeguarding meant the team had to devise their own criteria and scoring system. Although the study team was able to draw on many years of professional experience, it is possible that important factors were omitted. The scoring and weighting system used may skew some factors either negatively, i.e. not attaching enough importance to reporting, or positively giving too much weight to a factor. This study did not measure the fidelity of researchers’ actions to the policies set out by HEIs and it is possible that researchers in these HEIs do not adhere to their SOPs. As we discussed it could also be that researchers use their own experience and training on safeguarding, rather than following the policies. We did not visit the HEIs individually and therefore may be unaware of other practices and protocols which might address child maltreatment in research context, but which are not referred to in the SOP, such as hidden or obscured cultural practices known to those who work at an institution but not formally written into the policies. Additionally, we were aware that particular departments and faculties might have their own specially designed SOPs (e.g. the sports). These were collected and recorded where possible and addressed in the data analysis stage of the research. However, the researchers argue that university-wide SOPs are needed to address the full vulnerability of children in research, in addition to specific departments and institutions. The sample of HEIs is likely to be self-selecting; we did not use freedom of information request, we requested the SOP from each institution. In addition we only asked the highest ranked research universities. Thus it seems likely the sample in this study would give a more positive picture of safeguarding practices than if we had used a more compulsory approach and included all universities. The sample is only drawn from HEIs in the UK and may not be representative of practices in other countries. Finally, the researchers were aware that universities may have series of interlinking policies that address all issues, rather than one SOP. Some universities did indeed send multiple policies (for example, on the vetting and barring scheme). We argue however that there is a need for consistency and transparency so that children are best protected in research settings. The presence of multiple interlinking policies make it that much more difficult to understand the roles and responsibilities of researchers regarding children in research.

## Conclusions

Having a SOP which guides researchers in matters of child maltreatment may not make children safer in research. However, having clear and concise guidance in place can help reduce risk for children, researchers and HEI’s. The lack of coherent standard operating polices which guide researchers to relevant legal requirements and ethical guidance seems more likely to leave researchers unaware, and often unprepared, of the complex issues in even basic child protection practices (such as how to make a referral to local social work colleagues). While there are undoubtedly some highly experienced and well-educated researchers working on children’s research within these HEIs, there is a significant gap in many SOPs to prevent ill-informed and inexperienced researchers from putting children at risk or leaving children in abusive situations simply because they do not have the understanding to recognise abuse nor the skills to make appropriate referrals.

The findings of this study demonstrate that few HEIs appear to have considered child protection and child maltreatment issues in research. These institutions often have world leading research programmes that consider some of the most vital questions to the health and wellbeing of our children. There are two particular dangers. First, some children will remain in abusive situations when they could have been helped. Second, HEIs potentially expose themselves to avoidable scandal and notoriety. Consensus is needed on safer ways to conduct research with children and this should be reflected in policy and guidance for all researchers.

## Abbreviations

HEI: Higher Education Institution
SOP: Standard operating procedures
HESA: Higher Educational Statistical Agency
RAE: Research assessment exercise
